# INVESTIGATING DRUG-INDUCED DEMENTIA IN A CNS MPS ASSESSING DEFICITS IN LTP FROM ANTICHOLINERGIC COGNITIVE BURDEN

**DOI:** 10.1093/geroni/igae098.2981

**Published:** 2024-12-31

**Authors:** Kaveena Autar, Maria Grisales, Chelsea Honore, Xiufang Guo, David Morgan, Veronique Michaud, Jacques Turgeon, James Hickman

**Affiliations:** Hesperos, Clermont, Florida, United States; Hesperos, Orlando, Florida, United States; Hesperos, Orlando, Florida, United States; Hesperos, Orlando, Florida, United States; Michigan State University Grand Rapids, Grand Rapids, Michigan, United States; GalenusRx Inc, Orlando, Florida, United States; GalenusRx Inc, Orlando, Florida, United States; Hesperos, Orlando, Florida, United States

## Abstract

Microphysiological systems (MPS) provide an avenue for small-scale modelling of organ constructs. In conjunction with human induced-pluripotent stem cells (hiPSCs) cultured on microelectrode arrays (MEAs), MPS enable the exploration of functional deficits in concurrence with drug efficacy. Central nervous system (CNS) neural network MPS can evaluate compound-induced cognitive impairment via synapse-dependent, long-term potentiation (LTP). The present work employed a previously validated hiPSC-cortical neuron LTP model to investigate drug-induced dementia onset by Anticholinergic Cognitive Burden (ACB). Utilizing an established CNS model of LTP, we have successfully been able to evaluate a dose-response relationship between ACB drug concentration and cognitive deficits. After discovering that many ACB drugs cause clinical drowsiness, caffeine was employed to re-awaken neuronal activity, akin to increased vigilance. Donepezil, an acetylcholinesterase inhibitor commonly used in the treatment of Alzheimer’s disease (AD), confirmed that LTP deficits resulted from cholinergic neurotransmission, demonstrating the ability of our CNS MPS to assess mechanism-specific synaptic decline. Synergistic effects of ACB drugs and amyloid-beta42 oligomers, which agglomerate to produce neurotoxic plaques in AD, depicted an exacerbation of LTP deficits, suggesting a higher risk of cognitive decline in patients with early-onset AD using anticholinergics. This study illustrated the ability of our human, CNS-based MPS to recapitulate cognitive dysfunction preceded by neurotransmitter homeostatic imbalance, further validating our LTP model for investigating mechanism-dependent disease phenotypes. This model can be expanded to assess synergistic effects of drugs effecting neural network integrity on preexisting conditions causing cognitive impairment, which were observed to be translatable to clinical symptoms.

